# Gene Regulatory Network Inference and Gene Module Regulating Virulence in *Fusarium oxysporum*

**DOI:** 10.3389/fmicb.2022.861528

**Published:** 2022-06-02

**Authors:** Regnier Cano, Alexandre Rafael Lenz, Edgardo Galan-Vasquez, Jorge H. Ramirez-Prado, Ernesto Perez-Rueda

**Affiliations:** ^1^Centro de Investigaciones Científicas de Yucatán, Mérida, Mexico; ^2^Departamento de Ciências Exatas e da Terra, Universidade do Estado da Bahia, Salvador, Brazil; ^3^Departamento de Ingeniería de Sistemas Computacionales y Automatización, Instituto de Investigaciones en Matemáticas Aplicadas y en Sistemas, Universidad Nacional Autónoma de México, Ciudad Universitaria, Mexico, Mexico; ^4^Instituto de Investigaciones en Matemáticas Aplicadas y en Sistemas, Unidad Académica Yucatán Universidad Nacional Autónoma de México, Mérida, Mexico

**Keywords:** *Fusarium oxysporum*, virulence, gene regulation, regulatory networks, transcription factors, comparative genomics

## Abstract

In this work, we inferred the gene regulatory network (GRN) of the fungus *Fusarium oxysporum* by using the regulatory networks of *Aspergillus nidulans* FGSC A4, *Neurospora crassa* OR74A, *Saccharomyces cerevisiae* S288c, and *Fusarium graminearum* PH-1 as templates for sequence comparisons. Topological properties to infer the role of transcription factors (TFs) and to identify functional modules were calculated in the GRN. From these analyzes, five TFs were identified as hubs, including FOXG_04688 and FOXG_05432, which regulate 2,404 and 1,864 target genes, respectively. In addition, 16 communities were identified in the GRN, where the largest contains 1,923 genes and the smallest contains 227 genes. Finally, the genes associated with virulence were extracted from the GRN and exhaustively analyzed, and we identified a giant module with ten TFs and 273 target genes, where the most highly connected node corresponds to the transcription factor FOXG_05265, homologous to the putative bZip transcription factor CPTF1 of *Claviceps purpurea*, which is involved in ergotism disease that affects cereal crops and grasses. The results described in this work can be used for the study of gene regulation in this organism and open the possibility to explore putative genes associated with virulence against their host.

## Introduction

The species *Fusarium oxysporum* comprises a group of ubiquitous inhabitants of soils and plant pathogens causing vascular wilt and root diseases on a broad range of agricultural and ornamental plants worldwide. *F. oxysporum* can be divided into more than 120 formae speciales (*Fusarium* sp.) according to the pathogenicity to a set of host plants, and some formae speciales of *F. oxysporum* are further divided into several physiological races (Guo et al., 2014).

In recent years, gene regulatory networks (GRNs) have grown popular as an effective applied biology approach for describing relationships between regulatory components (transcription factors, or TFs) and their target genes, or TGs (e.g., enzymes and structural proteins), key components of cell circuits (Mercatelli et al., 2020). GRNs have been used to understand many global cellular processes, such as diseases, cell growth, and the improvement of omics data interpretation, including single-cell RNA sequencing (Jackson et al., 2020; Lenz et al., 2020). In this regard, a GRN is a collection of interactions represented in a graph, where genes or proteins are represented as vertices and their regulatory interactions are represented as edges. The network can be directed, where the interaction goes from the tail (*u*) to the head (*v*), or undirected, where there is no direction of the interaction.

To date, in the fungi scope, GRNs have extensively focused on *Saccharomyces cerevisiae* S288C (Guelzim et al., 2002; Lee et al., 2002; Pe'er et al., 2002; Segal et al., 2003; Nachman et al., 2004; Kim et al., 2006; Darabos et al., 2011; Jackson et al., 2020), *Neurospora crassa* OR74A (Hu et al., 2018), *Aspergillus nidulans* FGSC A4 (Hu et al., 2018), and *Fusarium graminearum* PH-1 (Guo et al., 2014). In contrast, the inference and analysis of GRNs for filamentous fungi remain incipient. In this regard, for *F. oxysporum* there have been no in-depth studies for the GRN. Therefore, it remains to be determined how cellular components work systemically to regulate *F. oxysporum* development, invasive growth, and virulence, among other processes.

In this context, *F. oxysporum* is a ubiquitous, soil-borne pathogen which causes devastating vascular wilt in more than 100 plant species and poses a serious threat to a wide range of economically important crops, such as banana, cotton, melon, and tomato (Gordon and Martyn, 1997). Thus, *F. oxysporum* represents a good fungal model to determine and expand the repertoire of genes associated with virulence mechanisms. In this regard, a network comprising interconnected and overlapping signaling pathways is activated once *F. oxysporum* recognizes a host in its vicinity. These pathways include mitogen-activated protein kinase signaling pathways (Di Pietro et al., 2001), Ras proteins and G-protein signaling components and their downstream pathways (Jain et al., 2002, 2005; Martínez-Rocha et al., 2008), components of the Velvet complex (LaeA/VeA/VelB) (López-Berges et al., 2013), and cAMP pathways (Jain et al., 2005), among others. The components of different pathways regulate expression of pathogenicity genes conferring virulence to *F. oxysporum*.

Therefore, in this work, based on a criterion of TF–TG orthology relationships of four related species with well-known regulatory interactions, combined with TF binding site (TFBS) predictions, we inferred the GRN for a reference strain, *F. oxysporum* f. sp. *lycopersici* 4287, a tomato pathogen. First, GRNs of related species (*A. nidulans, N. crassa, S. cerevisiae*, and *F. graminearum*) allowed the mapping of orthologous interactions. Further, TFBS predictions provided accuracy to TF–TG relationships. Finally, based on sequence comparisons between virulence factors deposited in the Database of Fungal Virulence Factors (DFVF) and the genes in *F. oxysporum*, we analyzed those genes associated with virulence and identified the most prominent functions associated with them. We consider that the GRN inference for this reference strain opens the opportunity to explore novel genes associated with virulence against hosts in a context of regulatory interactions.

## Data and Methodology

### Fungal Genomes Analyzed

Genomic data for *F. oxysporum* f. sp. *lycopersici* 4287 (GCA_000149955), *F. graminearum* PH-1 (GCA_000240135), *N. crassa* OR74A (GCA_000182925.2 NC12), and *S. cerevisiae* S288c (GCA_000146045.2 R64-1-1) were downloaded from the Ensembl Fungi server.^1^ Genomic data for *A. nidulans* FGSC A4 (s10-m04-r16) were downloaded from AspGD.^2^

### Identification of Orthologous Proteins

To identify orthologous proteins between *F. oxysporum* and model fungi (*F. graminearum*, *N. crassa*, *S. cerevisiae*, and *A. nidulans*), we used the program ProteinOrtho (v 6.0.15; Lechner et al., 2011) with an *E*-value of <1e-05, a sequence coverage of ≥ 50%, and minimal percent identity of best Blast hits of 25%, except for the report of singleton genes without any hit (Lechner et al., 2011). In brief, ProteinOrtho implements an extended version of the reciprocal best heuristic alignment (Lechner et al., 2011), reducing the amount of memory required for orthology analysis, compared to OrthoMCL and Multi-Paranoid, and the performance is comparable with that of OrthoMCL (Nichio et al., 2017).

### Identification of Transcription Factors

To assess the diversity of TFs, protein sequences of whole proteomes were used to search TF domains using InterProScan (v5.25–64.0; Jones et al., 2014). InterProScan was used to map interpro families and domains, based on the PFAM database. We used default parameters (hmmpfam –acc -A 0 –cpu 1 -E 0.01 -Z 350000). Afterwards, PFAM predictions of each species were collected making use of the 91 DNA-binding domains described in the catalog of the main eukaryotic TF families (Weirauch and Hughes, 2011), which was also used for the CIS-BP database.

### Upstream Sequences

DNA sequences comprising 1,000 bp upstream of each gene of *F. oxysporum* were extracted, considering the annotation in gff3 format and whole-genome sequences.

### Weight Matrices Used to Identify TFBSs

The weight matrices associated with TFs from *F. oxysporum* were obtained from the CIS-BP Database (Weirauch et al., 2014). A cross-validation was performed to check locus tag and gene name for each TF, crossing information from the reference genome and CIS-BP.

### TFBS Predictions

For each TF–TG interaction, TFBS prediction was carried out. RSAT matrix-scan (http://rsat.sb-roscoff.fr/) was used to predict the TFBSs by using all the respective position weight matrices (PWMs) from *F. oxysporum*, obtained from the CIS-BP Database (Weirauch et al., 2014). RSAT matrix-scan analyses were performed with “cis-bp” as matrix format. Other default parameters were maintained, including an *p*-value of < 1*e*-4 as the upper threshold.

### SQLite3 Database

All the information concerning the GRN was organized in a SQLite database by modeling six tables: “gene,” “ortho,” “pwm,” “regulation,” “tfbs_prediction,” and “network_node.” Input data obtained in the previous steps were inserted in the tables: “gene,” “ortho,” “pwm,” and “regulation” (these data are available as Supplementary Material S1).

### Inference of the Gene Regulatory Network

To reconstruct the GRN of *F. oxysporum*, the interactions with experimental evidence collected in four model organisms were used: *S. cerevisiae* with 6,709 nodes and 179,601 interactions (Monteiro et al., 2020); *A. nidulans* with 5,969 nodes and 10,018 regulatory interactions; *N. crassa* (7,446 nodes and 20,499 regulatory interactions; Hu et al., 2018), and *F. graminearum* (13,153 nodes and 39,459 regulatory interactions; Guo et al., 2014). In this regard, the inference is based on the hypothesis that orthologous TFs generally regulate the expression of orthologous TGs (Yu et al., 2004; Galán-Vásquez et al., 2016).

The orthology relationships can be defined as 1:N, N:1 or N:N, found by ProteinOrtho were defined as all-to-all by our tool. For instance, the protein AN5067 of *A. nidulans* has a 1:N orthology relationship; so it generated two entries in our orthologs database:

AN5067 FOXG_11784.
AN5067 FOXG_15825.

However, the absolute majority of orthology relationships found were 1:1, as shown below:

*F. graminearum* 260 of 9,302.
*A. nidulans* 673 of 5,763.
*N. crassa* 383 of 5,649.
*S. cerevisiae* 372 of 2,143.

Therefore, the orthology of TFs and TGs considering the GRNs previously characterized in the model organisms was mapped, if both elements were identified in *F. oxysporum*. In addition, we reinforced the assignment with TFBS predictions.

### Virulence Proteins

A total set of 2,058 proteins related to virulence were downloaded from the DFVF (Lu et al., 2012). The DFVF contains information about 2,058 pathogenic genes expressed by 228 fungal strains from 85 genera. Based on these proteins, we identified by orthology the virulence protein-encoding genes within the GRN of *F. oxysporum*. The program ProteinOrtho (v 6.0.15) was used with parameters as previously described.

## Results and Discussion

### Prediction of TFs in *Fusarium oxysporum*

To identify those proteins devoted to regulation of gene expression in *F. oxysporum* (GCA_000149955), the repertoire of TFs was identified by Pfam assignments and considering a dataset of 91 Pfam IDs associated with TFs described in the catalog of the main eukaryotic transcription factor families (Weirauch and Hughes, 2011), also used by the CIS-BP database. From these assignments, 503 proteins were identified as TFs; in other words, 2.3% of the total proteins (17,696) that *F. oxysporum* contains are associated with gene regulation. These proteins are classified into 39 different families, where the Fungal Zn(2)–Cys(6) binuclear cluster domain (PF00172) is the more abundant, with 264 members, followed by Zinc finger, C2H2 type (PF00096) with 67 members is the most abundant family of TFs, and Helix–loop–helix (HLH) DNA-binding domain (PF00010) with 37 members. These three families enclosed 73.1% of the proteins identified in *F. oxysporum*, whereas 36 families contributed to 26.9% of the total repertoire of TFs. From a functional perspective, members of the Zn(2)–Cys(6) family regulate diverse cellular processes, such as sugar and amino acid metabolism, cell cycle, and nitrogen utilization, which are among the most crucial processes for survival (MacPherson et al., 2006). Indeed, zinc cluster TFs exhibit diverse regulatory roles, can have overlapping functions (Shelest, 2008), and include a high number of proteins with experimental evidence in fungi (Shelest, 2017).

The HLH family contains proteins that regulate cellular differentiation, and morphogenesis and metabolism in *Candida albicans* (Doedt et al., 2004), developmental complexity in filamentous fungi (Dutton et al., 1997), and regulation of the cell cycle (Whitehall et al., 1999), among others. In addition, some members of this family are involved in determining virulence [such as Efg1p of *C. albicans* (Stoldt et al., 1997)], suggesting the versatility of regulatory roles they are involved in.

### Gene Regulatory Network

The GRN in *F. oxysporum* was inferred after considering orthology information from curated regulatory interactions of fungal models: *A. nidulans*, *N. crassa*, *S. cerevisiae*, and *F. graminearum*. When orthologues of a TF–TG relationship in a model organism were identified for both the TF and TG in *F. oxysporum*, a regulatory interaction was established (Yu et al., 2004; Galán-Vásquez et al., 2016). This inference is based on the hypothesis that if the sequences corresponding to TFs and TGs are conserved in the model organisms and in *F. oxysporum*, then regulatory interactions are also conserved. Similar approaches have been proposed for *Penicillium echinulatum* 2HH, *Penicillium oxalicum* 114–2, and *Ustilago maydis* (Lenz et al., 2020; Soberanes-Gutiérrez et al., 2021).

**Figure 1:**
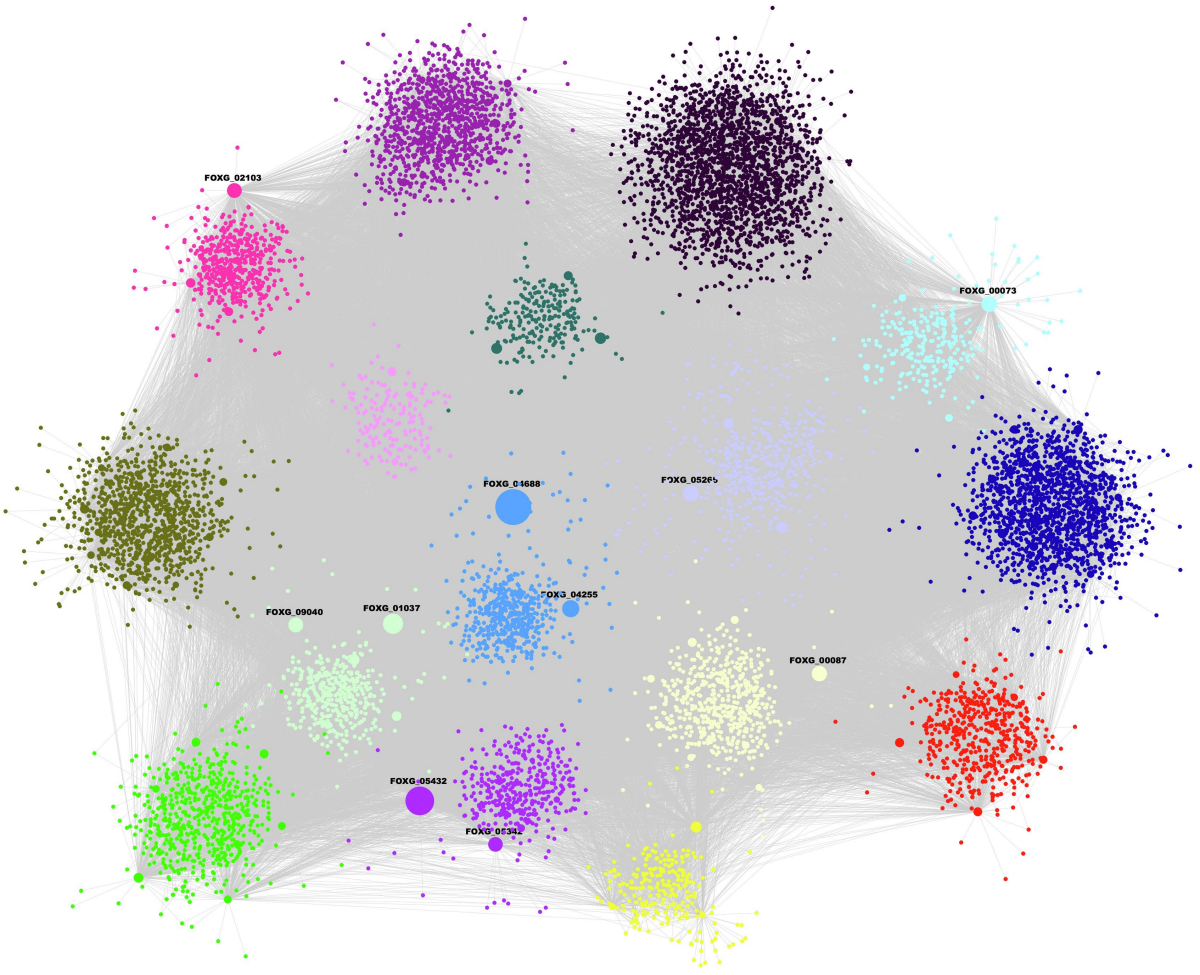
Gene regulatory network (GRN) of *Fusarium oxysporum*. Each color represents a different community and the size of the node is proportional to the output degree. Ten TFs are plotted as highly connected nodes, and these regulate 50.28% of the network nodes.

The inferred network contains 10,128 nodes and 43,572 edges, and covers 57.23% of the total proteins that *F. oxysporum* encodes (Figure 1; Supplementary Table S1). In this GRN, 184 TFs of 503 predicted were included, and they were associated with 10,125 TGs. In topological terms, the GRN has an average degree of 4.3, and 20 self-loops. In this regard, the fungal_trans domain-containing protein, FOXG_04688, was identified with 2,404 target genes, the maximum “out degree” identified in the network. This protein contains a zinc finger domain and it is homologous to fungal regulatory proteins associated with sucrose utilization in the Ascomycota *Tolypocladium paradoxum* and to the maltose fermentation regulatory protein MAL13 of *Metarhizium anisopliae.* The Zn–Cys binuclear cluster DNA-binding domain consists of two helices organized around a Zn(2)Cys(6) motifs and binds to sequences containing two DNA half-sites composed of three to five C/G combinations, whereas three proteins, alcohol dehydrogenase (FOXG_12790), glutamate dehydrogenase (FOXG_01626), and non-reducing end alpha-L-arabinofuranosidase (FOXG_02500), were found with the maximum “in degree” (regulated by 24 TFs each).

In functional terms, the GRN has 8,650 interactions inferred as activators, 6,051 as repressors, and 28,871 with no evident regulation. Based on TFBS assignments, 2013 of 43,572 had one TFBS associated, 436 had two TFBSs, and 105 had three or more binding sites. The regulatory interactions inferred were preferentially assigned from *S. cerevisiae* (1,425 interactions), *N. crassa* (14,109 interactions), *A. nidulans* (3,548 interactions), and *F. graminearum* (24,789 interactions; Table 1).

**Table 1:** GRN of five species (four fungal models and *F. oxysporum*).

	*F. oxysporum*	*S. cerevisiae*	*A. nidulans*	*N. crassa*	*F. graminearum*
Nodes	10,228	6,709	5,969	7,446	13,153
Edges	43,572	179,601	10,018	20,499	39,459
Average degree	4.3	26.77	1.67	2.75	3.0

### Topological Properties of the GRN

In order to evaluate the global structure of the GRN of *F. oxysporum* and compare it against the fungal models, the topological properties were calculated for the five organisms. From this comparison, we found that *F. oxysporum* contains the second highest number of nodes in comparison to the other organisms, with 10,228, whereas *S. cerevisiae* contains the greater number of edges (179,601). When we compared the average degree, *S. cerevisiae* also contained the highest number in all the genomes (26.77), whereas *F. oxysporum* had 4.3, the second highest in comparison with the other organisms. This discrepancy could be associated with the fact that *S. cerevisiae* is the most studied yeast species.

In this regard, the highest clustering coefficient, or 1, indicates that nodes whose neighbors are connected between them form complete graphs. In the GRN of *F. oxysporum*, we identified 50 nodes (representing 0.49% of the network) with a clustering coefficient of 1, indicating that there are substructures such as triangles or more complex motifs; this is consistent with previous reconstructed networks (Lenz et al., 2020; Soberanes-Gutiérrez et al., 2021). On the other hand, 4,542 nodes (44.84% of nodes in the network) have a clustering coefficient equal to 0, whereas 1791 of the nodes in the network have a degree of 1 and 2. We also found a mean 0.111 clustering coefficient for the network, indicating that neighbors have <⅓ connections among them. In this regard, the nodes with the highest clustering coefficient indicate that there are small highly connected groups, suggesting the existence of a modularity in the network. This modularity enables the identification of groups of genes that can work independently in an organism’s biological process (Resendis-Antonio et al., 2005; Martínez-Antonio et al., 2008).

In addition, we identified the top five most important nodes, based on their connectivity and on the shortest paths between each pair of nodes (Table 2). In this regard, FOXG_04688, which codes for a zinc finger domain-containing protein, was found to be the most important node in degree centrality (0.2382739). This TF was also identified with the maximum out degree (see previous section).

**Table 2:** Centralities of top 5 nodes.

Level	Degree centrality	Closeness centrality	Betweenness centrality	Eigenvector centrality
1	FOXG_04688 (0.2382739)^*^	FOXG_14504 (0.00538830)	FOXG_04688 (0.001342)	FOXG_02500 (0.05238574)
2	FOXG_05432 (0.18485237)	FOXG_08042 (0.00537961)	FOXG_05432 (0.0010579)	FOXG_03418 (0.05093478)
3	FOXG_01037 (0.11987755)	FOXG_02500 (0.00537207)	FOXG_01037 (0.0009947)	FOXG_01626 (0.0498954)
4	FOXG_04255 (0.0987459)	FOXG_12790 (0.00530916)	FOXG_05265 (0.0009814)	FOXG_00928 (0.0498882)
5	FOXG_05265 (0.09045126)	FOXG_03680 (0.0052953)	FOXG_04255 (0.0009301)	FOXG_12790 (0.04926705)

* Between parentheses, the value of centrality of each gene is added, the higher the value, the more important the gene.

Furthermore, we identified that FOXG_14504 (cursive xynA; endo-1,4-beta-xylanase) is the node that minimizes the sum of distances to the other nodes, i.e., the node with the highest closeness score (0.00538830). FOXG_02500 (an alpha-L-arabinofuranosidase) is the node that interacts with other highly connected nodes, i.e., the node with the highest eigenvector centrality (0.05238574). From a functional perspective, FOXG_14504 is a central node regulated by 22 TFs, mainly associated with the Zn(2)–Cys(6) binuclear cluster domain, suggesting that these proteins regulate similar processes. FOXG_14504 is homologous to XynC of *Aspergillus fumigatus*, involved in degradation of plant cell wall polysaccharides, a central process in infection mechanisms (de Vries and Visser, 2001). In addition to the highest closeness centrality, the protein XynA (FOXG_14504; endo-1,4-beta-xylanase) is a xylanolytic enzyme involved in the degradation of xylan, the main component of hemicellulose. Efficient hydrolysis of hemicellulose is also supported by other enzymes which act synergistically, like FOXG_02500 (alpha-L-arabinofuranosidase), which has the highest eigenvector centrality. Hemicellulose constitutes about 30% of plant cell walls; consequently, hemicellulose degradation genes play a central role in the fungal nutrition strategy (Andlar et al., 2018) and infection mechanisms (de Vries and Visser, 2001), which can be observed by analyzing the centrality metrics in the *F. oxysporum* GRN.

FOXG_04688, a regulatory protein probably involved in maltose and sucrose metabolism (as described for its homologues in *S. cerevisiae*) was also found to be the most significant when the betweenness centrality of a node *v* (0.001342) was calculated. In this regard, the betweenness centrality of a node is defined as the sum of the fraction of all-pairs shortest paths that pass-through *v*, i.e., the influence of a vertex over the flow of information between every pair of vertices under the assumption that information primarily flows over the shortest paths between two vertices.

Finally, we identified that six proteins are in the top five having more than one centrality: FOXG_04688, FOXG_05432, FOXG_01037, FOXG_01037 in degree and betweenness centrality; and FOXG_02500 and FOXG_12790 in closeness and eigenvector centrality. These proteins have been described as serine/threonine-protein kinase PLK1-like, Mannose-1-phosphate guanylyltransferase, methyl-accepting chemotaxis protein; K03776 aerotaxis receptor, bZIP domain-containing protein, non-reducing end alpha-L-arabinofuranosidase, and alcohol dehydrogenase, respectively. Therefore, these proteins are important for monitoring and transmitting information within the network, i.e., they can be affected quickly by changes in any part of the network and can modulate expression changes in other parts of the organisms.

At the structural level, these proteins can connect the different subunits of the network. Thus, to identify the most connected TFs associated with the reconstructed network, we identified the five top hubs (Table 3). A hub was defined as a TF with connections with many other nodes. From these hubs, the two most connected are the Fungal_trans domain-containing protein (FOXG_04688), which regulates 2,404 targets, and Mannose-1-phosphate guanylyltransferase (FOXG_05432), which regulates 1,864 genes.

**Table 3:** Top 5 identified hubs in the reconstructed network.

**Protein ID**	**Number of** **TGs**	**Function**	**Biological process (GO)**
FOXG_04688	2,404	Fungal-specific transcription factor domain	Metabolic process and single-organism process
FOXG_05432	1,864	Mannose-1-phosphate guanylyltransferase	Cellular process and response to stimulus
FOXG_01037	1,208	Cutinase gene palindrome-binding protein	Metabolic process and single-organism process
FOXG_04255	998	Conserved hypothetical protein	Organonitrogen compound biosynthetic process and organonitrogen compound metabolic process
FOXG_05265	910	Protein similar to bZIP transcription factor AtfA	Single-organism process

### Identification of Communities in the GRN

**Figure 2:**
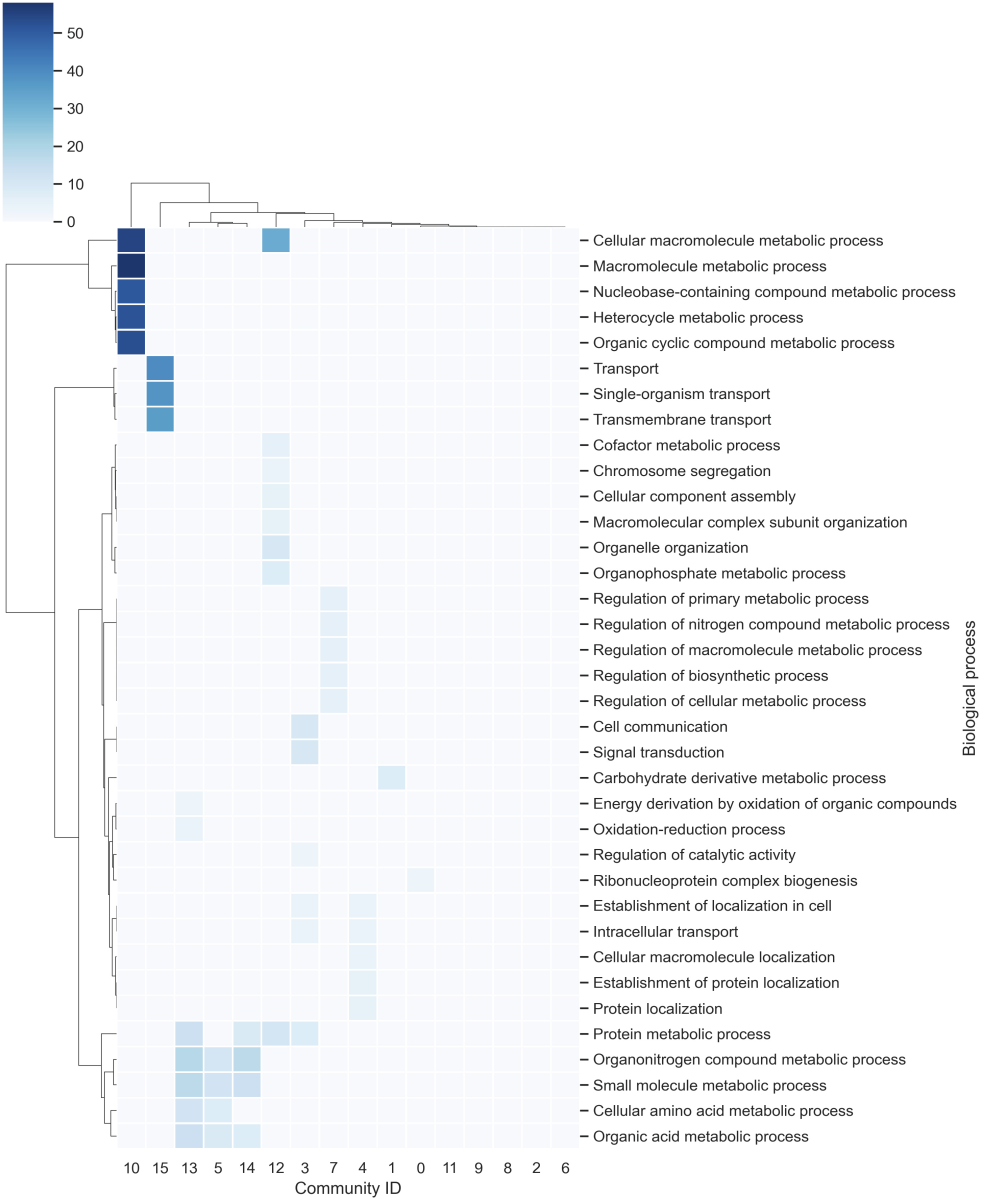
Communities in the network. The richest biological processes for each community were identified and hierarchically clustered based on Euclidean distance measures and Ward’s method for linkage analysis. Each row represents the gene ontology (GO) term for a biological process, and each column represents the community ID.

In order to identify the most related elements in the GRN, the inferred network was analyzed in terms of communities. To this end, a community was defined as a subset of nodes densely connected in comparison with the rest of the network (Radicchi et al., 2004). The communities were determined using the Blondel’s method, which allocates a new community to each node in the network and then moves a node to the community of one of its neighbors with whom it achieves the maximum positive contribution to modularity. This process is performed for all nodes until no further improvement is possible. Then, each community is treated as a separate node, and the process is continued until there is only one node remaining or the modularity cannot be raised in a single step (Blondel et al., 2008). Finally, these modules were functionally analyzed with gene ontology (GO) term enrichment. From this analysis, the *F. oxysporum* network contains 16 communities, where the longest contains 1,923 genes and the smallest contains 227 genes (Supplementary Table S2). In functional terms, the communities with the greatest diversity of enriched biological processes correspond to: Community-12, with a large proportion of genes related to protein metabolic processes and organelle organization, among others, with 23 proteins predicted as TFs; Community-13, with genes associated with organonitrogen compound metabolic processes and small molecule metabolic processes and contains 14 proteins predicted as TFs; and Community-3, which contains genes related to signal transduction and cell communication, with 13 proteins predicted as TFs. On the other hand, communities 2, 6, 8, 9, and 11 do not contain enriched biological processes, indicating a high functional diversity (Figure 2).

### Module of Virulence-Related Genes

In order to study the genes associated with virulence in *F. oxysporum*, protein sequences derived from the virulence factor database were used to identify their homologues in the genome of *F. oxysporum*. From this analysis, we identified 432 proteins probably involved in this process, which included genes related to extracellular metalloproteases, subtilisin-like serine protease, dipeptidyl-peptidase, and vacuolar aspartic endopeptidase, and two TFs experimentally characterized, among others. From these, 283 genes were included in a module with 467 regulatory interactions.

**Figure 3:**
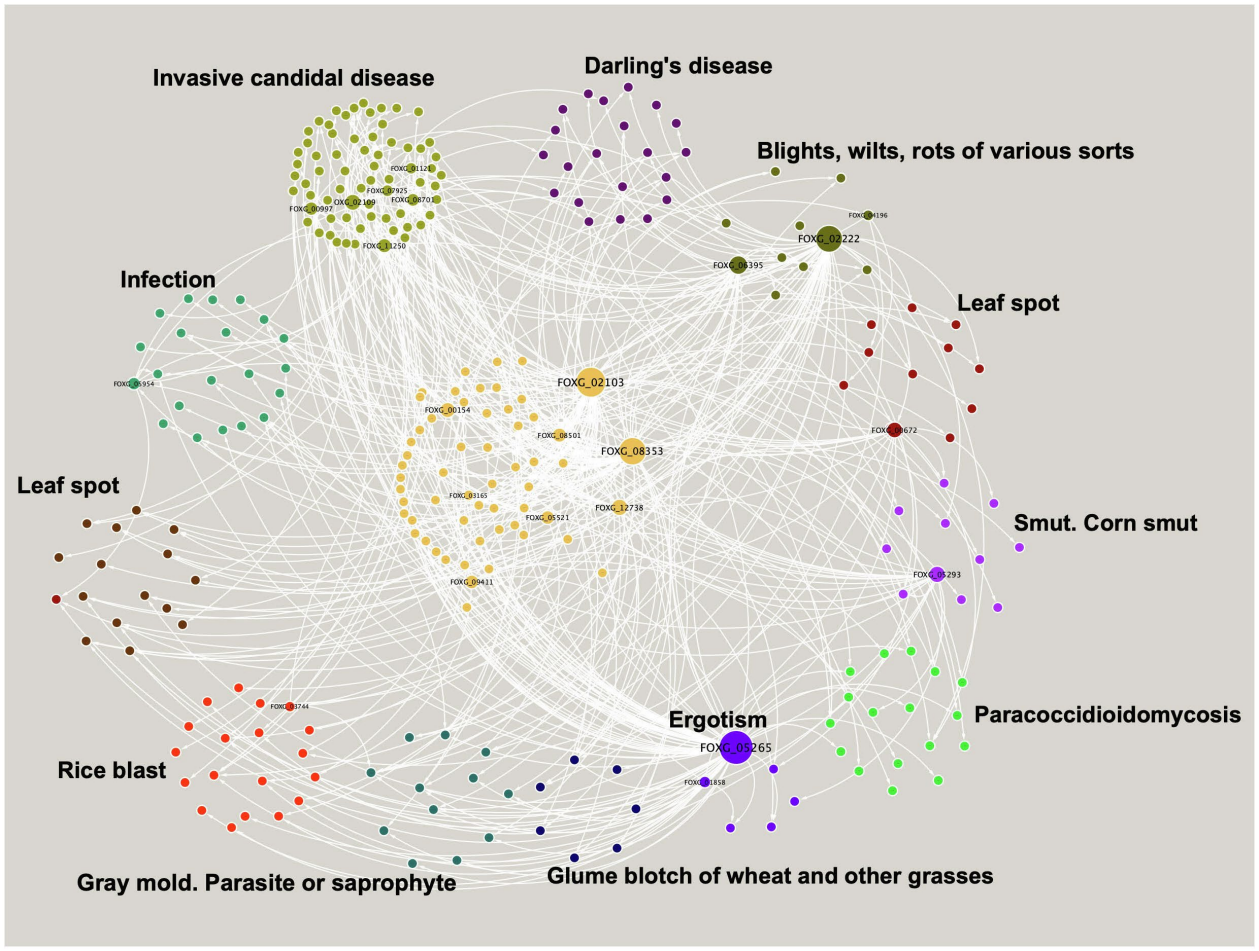
Virulence module in *F. oxysporum*. Nodes represent TFs and TGs, and the white edges represent regulatory interactions. The clusters show the most abundant diseases represented in the virulence module. The size of a node is proportional to its degree of output.

Topologically, the module consists of one giant component, with 10 TFs and 273 TGs, where the most highly connected node corresponds to a TF with a bZIP domain, FOXG_05265, which regulates 75 nodes that include 3 TFs and 72 TGs. This protein is homologous to the putative bZip transcription factor CPTF1 (Q8J0I5_CLAPU), involved in the ergotism disease caused by *Claviceps purpurea*, which affects cereal crops and grasses (Nathues et al., 2004; Figure 3).

On the other hand, six different TFs regulate expression of the Malate synthase gene, FOXG_03099, which is the most regulated gene in the virulence module and is described as a malate synthase involved in step two of the sub pathway that synthesizes (S)-malate from isocitrate. The protein is homologous to the malate synthase Mls1 (Q5J4D6_PHAND) of *Phaeosphaeria nodorum*, related to Glume blotch of wheat and other grasses disease (Lu et al., 2012).

In general, the genes associated with the virulence module are homologous to genes involved in 40 different diseases, such as leaf spot or ergotism (Figure 3), with a large cluster of 68 genes, mainly related to invasive candidal disease (mainly associated with cell communication, phosphate-containing compound metabolic process regulation of signaling, and response to stress, among others). As well as infection disease with 21 related genes (mainly exopeptidase, serine hydrolase, and carboxypeptidase activities), and Darling’s disease, with 21 genes related to chloroplast stroma and cell septum (Figure 3).

## Conclusion

The analysis described in this work, considers a guilt-by-association approach to infer the GRNs in *F. oxysporum*, based on TF–TG orthology relationships of four fungal species with well-known regulatory interactions. In a posterior step, the reconstructed network was evaluated in terms of its topological properties, identifying TFs as hubs, modules, and co-regulated genes. The predicted GRN was considering the orthology relationships identified with Proteinortho, a method that implements a blast-based approach to determine sets of (co-)orthologous protein sequences that generalizes the reciprocal best alignment heuristic (Lechner et al., 2011); and reinforced (when it was possible) with TFBS predictions.

We understand that the apparent absence of the accuracy of the method could open questions about the reliability of the predictions; however, all inferences were considering the TF–TG interactions with experimental evidences (included as Supplementary Material), such as *S. cerevisiae* (Monteiro et al., 2020), *A. nidulans* and *N. crassa* (Hu et al., 2018), and *F. graminearum* (Guo et al., 2014); whereas the orthology relationships were defined as 1:N, N:1 or N:N, found by ProteinOrtho. In this regard, the GRN was compared with Genomic feature of Fol4287 well documented by Ma et al. (2010), finding a high proportion of proteins identified in different functional groups previously described (between 30 and 92% of each dataset). Suggesting that our approach is able to identify those proteins associated with functional roles. In summary, we did not use expression data to infer the GRN, where diverse approaches to evaluate the accuracy of the method have been proposed. Instead, we used orthology inferences based on fungal models with well-known experimental evidence. Alternatively, an approach previously suggested to evaluate the accuracy of the GRN, would consider a probabilistic approach to estimate the functional coupling between genes, using the functional annotations from a gold standard set; however, this approach is useful to expand a well-known network with not considering sequence comparisons. In our case, if this approach is implemented to compare the real versus inferred networks, (at least) two challenges are found. The first one, the pair of TF–TGs used to infer the network in a new genome, would exhibit functional similarity to the pair of genes used as reference; the second one, we must assume that TF–TG used as a reference, exhibit functional congruency for large datasets, inclusive for TFs with multiple targets.

Since a functional perspective, the inference of the GRN of *F. oxysporum* provides an excellent opportunity to understand how genes and functional processes are interrelated in this organism. The GRN was analyzed in terms of its topological properties to infer the role of TFs in the context of the GRN and to identify functional modules. From these analyses, FOXG_04688 and FOXG_05265, which regulate 7,384 target genes, were identified as hubs. In addition, 16 communities were identified in the GRN, where the longest contains 1,923 genes and the smallest contains 227 genes. Finally, the module of virulence with 467 regulatory interactions identified a giant module with 10 TFs and 273 TGs, where the most highly connected node corresponded to the TF FOXG_05265 with a bZIP domain. Besides genome-wide approaches, targeted analysis of regulatory regions can elucidate regulatory divergence after speciation. For example, analyzing TF binding specificity of the transcription factor LEAFY homologues from different plant species, mosses, and algae, among others, revealed subtle changes in their preferred TFBS motifs, suggesting that the DNA binding specificity of this TF changed during land plant evolution (Sayou et al., 2016). Thus, the combination of numerous TFBS models with novel genome sequences could ultimately unlock mechanisms of GRN evolution. In this context, the inference of GRN described in this work can be improved with experimental data, such as the ChIP-seq and prediction of TFBS by phylogenetic footprinting studies, among others. In this regard, comparative ChIP-seq studies have identified a highly conserved TFBS motif for two TFs, but highly divergent binding events on conserved genes of different species (Schmidt et al., 2010). Therefore, we do not only consider that the inference of GRN is central to understanding the general topology of the network, but is the base stone to complement experimental studies to understand the network dynamics, and open the possibility to explore putative genes associated with virulence against their host.

## Data Availability Statement

The original contributions presented in the study are included in the article/Supplementary Material; further inquiries can be directed to the corresponding author.

## Author Contributions

All authors listed have made a substantial, direct, and intellectual contribution to the work and approved it for publication.

## Funding

This work was supported by Dirección General de Asuntos del Personal Académico-Universidad Nacional Autónoma de México (IA201221 and IN-209620) and CONACYT (320012; INFR-2016-01-269833).

## Conflict of Interest

The authors declare that the research was conducted in the absence of any commercial or financial relationships that could be construed as a potential conflict of interest.

## Publisher’s Note

All claims expressed in this article are solely those of the authors and do not necessarily represent those of their affiliated organizations, or those of the publisher, the editors and the reviewers. Any product that may be evaluated in this article, or claim that may be made by its manufacturer, is not guaranteed or endorsed by the publisher.

## Footnotes

^1^ https://fungi.ensembl.org/

^2^ http://www.aspgd.org
