# Supplementary material for: Gene Regulatory Network Inference and Gene Module Regulating Virulence in Fusarium oxysporum
Source: Front Microbiol. 2022 Jun 2;13:861528. doi: 10.3389/fmicb.2022.861528 (PMC9201490; doi:10.3389/fmicb.2022.861528)
Supplement: Supplementary file 3 [file Table_3.DOCX]

Table S3. Description of GRN of the fungal models.

*S. cerevisiae* (<https://doi.org/10.1093/nar/gkz859>)

The YEASTRACT+ portal gathers documented regulatory associations between transcription factors (TF) and target genes of yeast species. All available information on transcriptional associations were published in peer-reviewed international journals. The underlying experimental evidence was collected and classified as either DNA Binding or Expression Evidence. DNA Binding Evidence refers to experimental data obtained from chromatin immunoprecipitation (ChIP), ChIP-on-chip, ChIP-seq and electrophoretic mobility shift assay studies. Expression Evidence refers to data obtained from the comparative analysis of gene expression changes occurring in response to the deletion, mutation or over-expression of a given TF, based on reverse transcriptase-polymerase chain reaction, microarray analysis, RNA sequencing or expression proteomics. Based on the gathered Expression Evidence each regulatory association was classified as positive or negative, considering TFs that act as activators or repressors in these conditions, respectively. Based on this classification, YEASTRACT+ contains a total of 45 209 regulatory associations based on DNA-binding evidence and 161 783 on expression evidence.

*A. nidulans* and *N. crassa* (https://doi.org/10.3389/fmicb.2018.00027)

To collect research papers containing information on TF–target interactions, we searched articles in PubMed with the queries “(nidulans[Title/Abstract]) AND transcription [Title/Abstract],” “(crassa[Title/Abstract]) AND transcription [Title/Abstract]” and their variants (e.g., replacing “transcription” by “transcriptional”). In addition, we retrieved the lists of predicted TFs in the two species from FTFD, a database computationally predicting fungal TFs at genome scale. For A. nidulans, the curated papers associated with each TF in the database AspGD were also collected. For N. crassa, we directly searched articles in PubMed with the names of characterized TFs. In total, more than 1,000 papers were collected and checked for the presence of information on TF–target interactions.

For each regulatory interaction, the ID and name (if applicable) of TFs and targets, the type of experimental evidence, the regulatory function (repression or activation), and the PubMed ID of reference literature were recorded. Perturbations of TFs through gene deletion, knock-down of gene expression, protein inactivation, or similar manners were recorded as “TFdown” manipulations, while those through gene overexpression or introduction of gain-of-function mutants were classified into “TFup” manipulations. In TF-perturbation studies, the gene encoding the perturbed TF is often included in the differentially-expressed gene set in transcriptome data, but should not be considered as the target of its product.

*F. graminearum* (https://doi.org/10.1111/nph.13912)

F. graminearum GRN was inferred by adapting MinReg, a machine‐learning algorithm based on a BN model (Pe'er et al., 2002) which treats the expression level of each gene as a random variable and attempts to estimate the structural features of the dependencies in their joint probability distribution from the data. To collect sufficient expression data for reliable network prediction, we generated 27 expression profiles based on nine experiments (see the Materials and Methods section) (Table S1) and acquired 166 transcriptomic datasets curated at the Plant Expression database (PLEXdb) (Dash et al., 2012). Expression data from both sources were combined and normalized, generating a global gene expression data matrix as input data (Table S2).
